# Effect of pegylated phosphatidylserine-containing liposomes in experimental chronic arthritis

**DOI:** 10.1186/s40360-015-0022-0

**Published:** 2015-09-22

**Authors:** Paulo CM Urbano, Vanete T. Soccol, Vivian N. Teixeira, Patrícia G. Oliveira, Lidiane I. Filippin, Wagner H. Bonat, Carolina de Oliveira, Gustavo R. Rossi, Ricardo M. Xavier, Valderilio F. Azevedo

**Affiliations:** Biotechnology and Bioprocess Engineering, Universidade Federal do Paraná, Curitiba, Paraná Brazil; Rheumatology Department, Hospital de Clínicas de Porto Alegre (HCPA), Rio Grande do Sul, Brazil; Statistical Laboratory (LABEST), Universidade Federal do Paraná (UFPR), Curitiba, Paraná Brazil; Department of Cell Biology, Research Laboratory of Inflammatory Cells and Neoplastic, Universidade Federal do Paraná (UFPR), Curitiba, Paraná Brazil; Rheumatology Service and Internal Medicine, Hospital de Clínicas de Curitiba, Universidade Federal do Paraná (UFPR), Rua Alvaro Alvin, 224 casa 18, Curitiba, Paraná 80440080 Brazil

## Abstract

**Background:**

Phosphatidylserine-containing liposomes (PSL) have been shown to reduce inflammation in experimental models of acute arthritis, by mimicking the apoptotic process. The aim of this study was to evaluate the effect of pegylated PSL (PEG-PSL) on chronic inflammation of collagen induced arthritis (CIA) in DBA/1J mice.

**Methods:**

CIA was induced in 24 DBA/1J mice (*n* = 6/group), which were divided into control (0.9 % saline) or treated with PEG-PSL (5, 10 and 15 mg/kg/day, subcutaneously for 20 days). Clinical score, limb histology and measurement of cytokines in knee joints of animals by ELISA and cytometric bead array (CBA) were evaluated. The *in vitro* study employed macrophage cultures stimulated with 100 ng/ml of LPS plus 10 ng/ml of PMA and treated with 100 μM PEG-PSL.

**Results:**

Resolution of the disease *in vivo* and the inflammatory process *in vitro* were not observed. PEG-PSL, in doses of 10 and 15 mg/kg, were not shown to reduce the score of the disease in animals, whereas with the dose of 5 mg/kg, the animals did not show the advanced stage of the disease when compared to the controls. The PEG- PSL 5, 10 and 15 mg/kg treatment groups did not show significant reduction of TNF-α, IL-1β, IL-6, IL-2 and IFN-γ when compared to the controls. Disease incidence and animal weights were not affected by treatment. Regarding the paw histology, PEG-PSL did not yield any reductions in the infiltrating mononuclear, synovial hyperplasia, extension of pannus formation, synovial fibrosis, erosion of cartilage, bone erosion or cartilage degradation. The concentration of 100 μM of PEG-PSL has not been shown to reduce inflammation induced by LPS/PMA in the *in vitro* study. Treated groups did not show any reduction in inflammatory cytokines in the knee joints of animals affected by the disease compared to the control, although there were higher concentrations of TGF-β1 in all experimental groups.

**Conclusion:**

The experimental model showed an expression of severe arthritis after the booster. TGF-β1 as well other pro inflammatory cytokines were presented in high concentrations in all groups. PEG-PSL had no impact on the clinical score, the histopathology from tibial-tarsal joints or the production of cytokines in the knee joints. Other alternatives such as dosage, route of administration, and as an adjunct to a drug already on the market, should be evaluated to support the use of PEG-PSL as a new therapeutic tool in inflammatory diseases.

## Background

Phosphatidylserine (PS) is a phospholipid found in the inner part of the plasma membrane of viable cells [1]. In the process of apoptosis, PS is externalized and presented as a phagocytic signal for macrophage-mediated removal of apoptotic bodies, contributing to the homeostasis of the organism [1–5].

However, it is not only the organism that enjoys the benefits of PS. Some protozoa mimic the exposure of PS to be phagocytosed and begin the process of infection in macrophages, mainly by inhibiting nitric oxide and stimulating TGF-β1 and IL-10 [6–8]. Toxoplasma gondii [6, 8], Leishmania ssp [7, 9, 10], and trypomastigote forms of Trypanosoma cruzi [8], frequently employ this strategy. There are also reports of tumoral cells which excessively exhibit PS to promote immunosuppression of the immune system, allowing their development without interference from the organism [11].

Given these facts, in recent years the use of PS liposomes (PSL) has been shown to reduce inflammation in models of acute experimental arthritis [12–14]. One possible explanation for such an effect is that the apoptotic mimicking effect is triggered by a linking between PS and T cell immunoglobulin mucin receptors (TIM-1 and TIM-4) [2, 15, 16] and other receptors [17], mainly present in macrophages, resulting in phagocytosis of the vesicles and promotion of an anti-inflammatory response through the production of TGF-β and IL-10 [18].

Huynh et al. [18] were pioneers in demonstrating the reduction of *in vivo* inflammation using PSL, and still suggest that TGF-β is fundamental in the anti-inflammatory response.

Other studies have used PSL as an inhibitor of inflammation in carrageenan-induced arthritis model in mice (100 mg/kg, intraperitoneal) [12], for ischemia prevention in mice (5 mg/kg) [19], as an inhibitor of immunogenicity of recombinant molecules [20–22], for myocardial infarction in mice (5 mg/kg)[23] and even in magnetic resonance imaging (MRI) to evaluate the delivery routes of encapsulated drugs in mice, in addition to the use of pegylated PSLs (PEG-PSL) to maximize the anti-inflammatory effects [24]. Moreover, the consistent findings of Wu et al. [13] and Ma et al. [14] showed the intervention of PSL (5 mg/kg, intramuscular) in experimental arthritis in rats by inhibiting inflammation and bone loss in rats with adjuvant-induced arthritis.

Still unknown is the effect of PSL in the model of bovine collagen-induced chronic arthritis (CIA). The structural lesions in CIA are most analogous to human rheumatoid arthritis (RA), thus an excellent option for pre-clinical drug studies [25]. CIA depends on adaptive and humoral immunity (T and B cells) and the complement system for disease induction [26, 27].

In light of the possibility of mimicking apoptosis and reducing the inflammatory process, the present study evaluated the effect of PEG-PSL in the CIA model, to evaluate the therapeutic potential of PEG-PSL in the chronic phase of experimental arthritis in order to outline future applications in human autoimmune diseases.

## Methods

### Ethical aspects

All experiments were performed according to the Guiding Principles for Research Involving Animals (NAS) and approved by the Committee of Research and Ethics in Health of the Hospital de Clínicas de Porto Alegre (Porto Alegre, Brazil).

### Materials

Phosphatidylcholine (PC), PS and 1.2 diesterol-sn-glycero-3-phosphatidylethanolamine-N- (polyethylene glycol)-2000 (DSPE-Peg2000) were obtained from Avanti Polar Lipids, Alabaster, AL, USA. The type II (CII) bovine collagen was acquired from Chondrex, Redmond, WA, USA. Freund’s complete adjuvant (CFA), E. coli 0111:B4, phorbol myristate acetate (PMA) and protease inhibitor cocktail (P1860) were purchased from Sigma, St Louis, MO, USA. Inactivated *mycobacterium tuberculosis* strain H37RA was obtained from Difco, Detroit, MI, USA. The RAW 264.7 (ATCC® TIB-71™) macrophage strain, fetal bovine serum (FBS) and DMEM (Dulbecco’s Modified Eagle Medium) were obtained from *Gibco*®, Grand Island, NY, USA. Isoflurane was obtained from Abbott, Chicago, IL, USA. The *BD Cytometric Bead Array (CBA) Mouse Th1/Th2/Th17 Cytokine Kit* and the *Mouse IL-1β ELISA Set Kit* were obtained from Becton Dickinson, Franklin Lakes, NJ, USA. The *Mouse TGF-β1 Platinum ELISA* Kit was obtained from eBioscience, San Diego, CA, USA.

### Liposomes

Liposomes composed of PC, PS and DSPE-PEG2000 in a molar ratio of 68:30:2, respectively, were produced by *Encapsula Nanosciences* (Brentwood, TN, USA) following the process described below.

The phospholipids were added in the desired molar ratios in chloroform/methanol (90:10) and then were dried in an atmosphere of nitrogen. They were then suspended in PBS (pH7.4) in the transition temperature of the phospholipids and then underwent extrusion with porous polycarbonate membrane, measuring approximately 100 nm. The suspension was passed through the membrane 11 times until the formation of unilamellar liposomes with a diameter of 100 nm (Zetasizer Nano ZS90, Malvern, Westborough, MA, USA). Liposomes were stored in flasks with PBS (pH7.4) degassed at 4 °C and purged with argon. Under these conditions the effects of hydrolysis and oxidation are minimized.

### Animals

Female DBA/1j mice from 8 to 12 weeks of age were used. The animals were reared at ± 20 °C, with 12-h light/dark cycles and free access to food and water.

### Immunization protocol for bovine collagen type II mice

Polyarthritis by CIA in mice was induced according to the methodology proposed by Brand et al. [27].

Bovine type II collagen (CII) was dissolved in 0.1 M acetic acid at 4 °C for 12 h (2 mg/ml). CFA supplemented to 4 mg/ml of inactivated Mycobacterium tuberculosis (strain H37RA) was used [27]*.* CII (2 mg/mL) and CFA (4 mg/mL) were mixed in equal volumes to form an emulsion. CIA was induced by CII on day zero, via intradermal immunization (id) of 50 μL of emulsion at the base of the tail. On the 18th day, the mice received a booster in another part of the tail, following the same protocol, but with the incomplete Freund adjuvant (IFA), id. The animals were given Isoflurane anesthesia by inhalation for the immunizations and euthanize.

Finally, at the end of treatment, animals were euthanized by cervical dislocation. The knees were cut and preserved at −80 °C for cytokine analysis of the joint, while the legs were preserved in formalin for histological analysis.

### Treatment

The treatments with PEG-PSL 5, 10 and 15 mg/kg/day were started on the same day as the booster*.*

The CIA animals were divided into four experimental groups (*n* = 6/group): treatment vehicle (saline 0.9 %) daily subcutaneously (sc) or PEG-PSL 5 mg/kg/day sc; PEG-PSL 10 mg/kg/day sc; and PEG-PSL 15 mg/kg/day, sc. Either PEG-PSL or vehicle 100 μL were applied subcutaneously in the neck area for twenty days following the booster.

The doses used in this study were employed based on studies using PSL in an acute inflammatory arthropathy model [13, 14].

#### Clinical score

A blinded observer performed subjective analysis of the clinical severity score during the treatment period. The degree of swelling of the anterior and posterior legs of the animals was evaluated using a scale of 0 to 4 for each limb. To assess the progression of the disease in an animal, a scale of 0 to 16 was used, using the sum of the scores of all four limbs of each animal.

### Histological analysis

The tibiotarsal joints of the DBA/1J animals were isolated and immersed in 10 % buffered formalin for fixation for 24 h. Next, the tissues were decalcified in 10 % trichloroacetic acid (TCA) for approximately 18 h. These tissues were dehydrated and embedded in paraffin blocks. Slices 6 μm thick were arranged on microscope slides. The slides were stained using hematoxylin and eosin technique for analysis of the following parameters: synovial inflammation: (five high-power magnification fields - HMF) were analyzed for the percentage of infiltrating mononuclear cells: 0- absent, 1- mild (1–10 %), 2- moderate (11–50 %), 3- severe (51–100 %); synovial hyperplasia: 0- absent, 1- mild (5–10 layers of cells), 2- moderate (11–50 layers), 3- severe (>20 layers); extension of pannus formation: 0- absent, 1- mild, 2- moderate, 3- severe; synovial fibrosis: 0- absent, 1- mild (1–10 %), 2- moderate (11–50 %), 3- severe (51–100 %); erosion of cartilage: 0- absent, 1- mild (1–10 %), 2- moderate (11–50 %), 3- severe (51–100 %); bone erosion: 0- none, 1- minor erosion/s observed only under HMF, 2- moderate erosion/s observed under low amplification, 3- serious transcortical erosion/s grave conforming to the previous description [28] and for the analysis of cartilage degradation, safranin-O staining was conducted. All slices were microscopically analyzed by two blinded observers, and the images were captured by digital camera [28].

#### Immunomodulation *in vitro* of PEG-PSL in macrophages stimulated with LPS/PMA

The macrophage cell culture (RAW 264.7 - ATCC® TIB-71™) was maintained in DMEM (Dulbecco’s Modified Eagle Medium) containing 4500 mg glycose/L, L-glutamine and phenol red at 37 °C and 5 % CO_2_. The medium was supplemented with 10 % fetal bovine serum (FBS), plus penicillin 1 U/mL and streptomycin 1 μg/mL. The cells were cultivated in 96-well culture plates with a density of 5 × 10^4^ per well.

The macrophages were stimulated with 100 ng/mL of lipopolysaccharide (LPS) and 10 ng/mL of PMA for 12 h prior to treatment (Table 1).

**Table 1:** Experimental groups in *in vitro* study

	Untreated	PBS	PEG-PSL	LPS + PMA	LPS + PMA + PBS	LPS + PMA + PEG-PSL
Dose	-	10 μL	10 μL (100 μM)	100 ng/mL 10 ng/mL	100 ng/mL 10 ng/mL 10 μL	100 ng/mL 10 ng/mL 100 μM
Treatment regimen	-	24 h	24 h	12 h	12 h 24 h	12 h 24 h

*LPS* Lipopolysaccharide; *PMA* phorbol myristate acetate; *PEG-PSL* pegylated phosphatidylserine-containing liposomes

The treatment with 100 μM of PEG-PSL lasted 24 h, after stimulation for 12 h with LPS/PMA. After culturing, the supernatant was recovered to measure the levels of cytokines which were quantified by cytometric bead array (CBA) and enzyme-linked immunosorbent assay (ELISA).

#### Evaluation of cytokine production - IL-1β and TGF-β1 by ELISA

For the quantification of IL-1β, the *Mouse IL*-*1β ELISA Set* Kit was used. For analysis of TGF-β1 levels, the *Mouse TGF*-*β1 Platinum ELISA* Kit *was used*.

For evaluation of the production of IL-1β and TGF-β1 in the *in vivo* study, the knee joints of the animals were homogenized by Polytron™ Homogenizers (*Kinematica Inc., Keyland Court Bohemia, NY, USA*) at 4 °C with sterile PBS (pH7.4) with protease inhibitor (*Protease Inhibitor Cocktail*).

The samples were centrifuged at 10,000 g per 10 min [28]. The supernatant was collected for analysis. For evaluation of production of IL-1β and TGF-β1 in the *in vitro* experiment, Supernatants of the macrophage cultures were harvested after the treatment and used in the assay. The methodology followed the kit manufacturer’s specifications.

#### Assessment of the Th1/Th2 and Th17 profile by CBA

The concentrations of IL-2, IL-4, IL-6, IFN-γ, TNF-α, IL-17A, IL-10 were measured, of the macerated tissue of the knee joints of the animals in the *in vivo* study and the supernatant of macrophage cultures of the *in vitro* experiment, with the *BD Cytometric Bead Array Mouse Th1/Th2/Th17 Cytokine Kit.* Data were obtained by flow cytometry using the BD *FACSCalibur* and analyzed using the *FCAP3*.0™ software (*Becton Dickinson,* Franklin Lakes, NJ, USA).

### Statistical analysis

For analysis of the clinical scores, the comparison of the groups was obtained using a nonlinear model, using the R software (*R Project for Statistical Computing* – Federal University of Paraná), with the following formula:

$$ \frac{A}{1+{e}^{-\left(x-xo\right)/5}} $$

Where:

A = Asymptote

-*(x-xo)/5* = Half-life

*5* = Scale

The results were described by value, standard error, t-value and *p*-value (≤0.05).

To assess the relationship between cytokine production *in vivo* and *in vitro* experimental groups, the Kruskal-Wallis test and later, the unpaired Wilcoxon test were used. For histological analysis, the Kruskal-Wallis test and Dunn’s Multiple Comparison test were used. GraphPad Pris software version 5.03 for Windows (*GraphPad Software, San Diego, CA, USA*) was used.

## Results

### Effect of PEG-PSL treatment on the animals’ clinical score

**Fig. 1:**
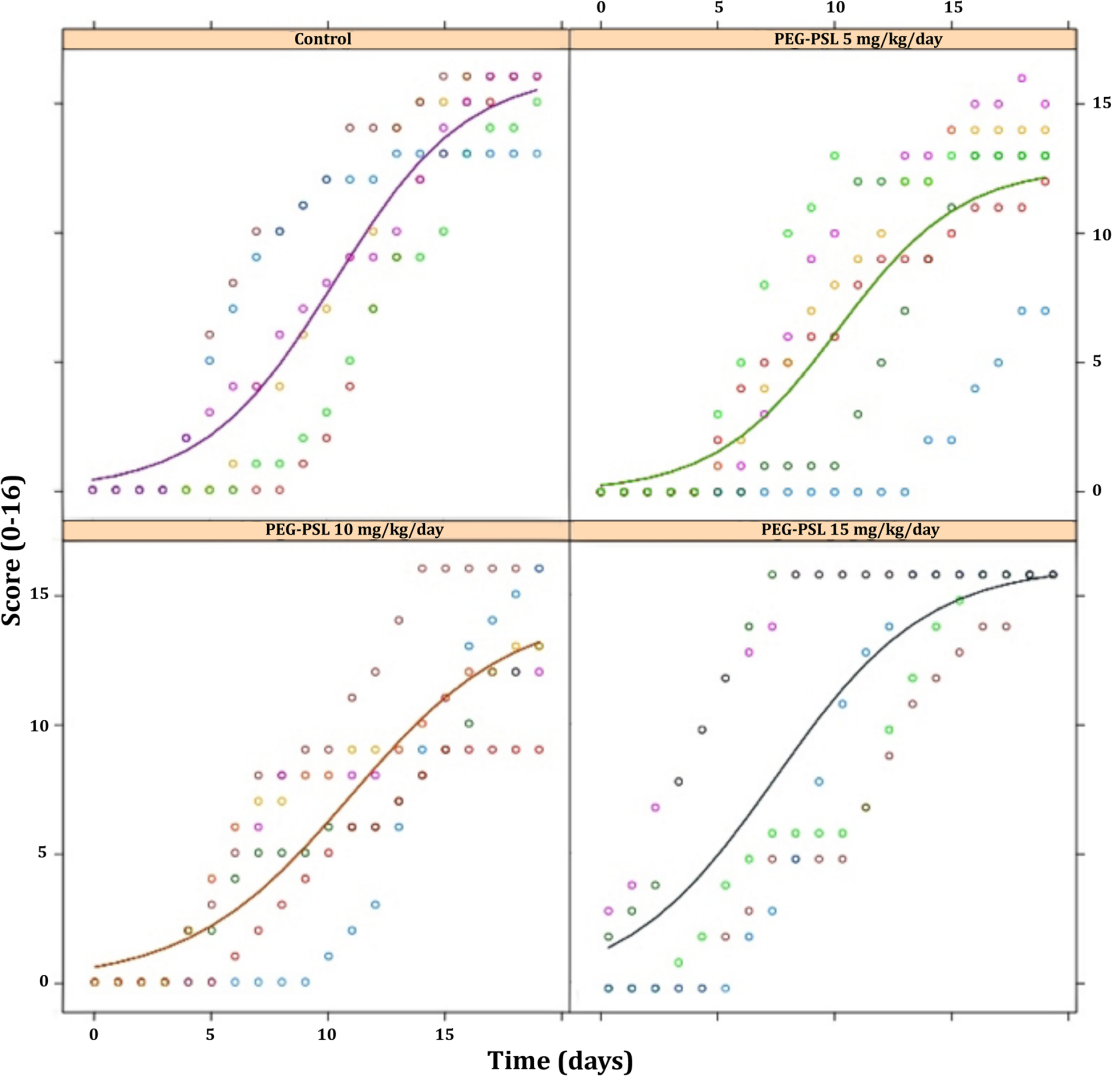
Clinical score of the limbs of animals in the experimental groups. The animals were monitored daily by analysis of clinical signs of arthritis as described by Brand et al. [27] using a score for severity, as follows: 0 = no evidence of erythema and swelling, 1 = erythema and mild swelling confined to the tarsals or ankle joint, 2 = erythema and mild swelling extending from the ankle to the tarsals, 3 = erythema and moderate swelling extending from the ankle to the metatarsal joints, 4 = erythema and severe swelling encompassing the ankle, foot, and digits, or ankylosis of the limb. The total score is the average of the scores of all four limbs of each animal from the onset of the disease. Each of the groups contained six animals. Each colored dot represents the trajectory of one animal during the 20 treatment days. Nonlinear statistical analysis method was used. Three factors were evaluated: asymptote (a), half-life (MED) and scale (S). The results were described by value, standard error, t-value and *p*-value (≤0.05) (see Table 2)

Figure 1 shows the dynamics of disease progression based on the evaluation of the daily clinical score. The trajectory of each individual is illustrated via the colored dots during the treatment period and shows some heterogeneity in the DBA/1J strain of mice used in this study.

Incidence of the disease was 100 %, evidencing a universal induction of arthritis in this study. By day 14 following the booster, all animals were afflicted.

Considering the length of treatment (20 days) and the daily administration of 5, 10 and 15 mg/kg of PEG-PSL, the results showed no significant difference in the reduction of disease progression as assessed by clinical score between the treatment groups (10 and 15 mg/kg) and the control group (Fig. 1). The group treated with PEG-PSL 5 mg/kg presented an upper asymptote of clinical score significantly less than the control, i.e., the animals of the group treated with 5 mg/kg did not show a greater degree of disease severity (Fig. 1 and Table 2), evidencing a possible effect on the delay of disease activity.

**Table 2:** Statistical analysis of the clinical score of experimental groups

Coefficients	Value	Standard error	t-value	p-valor
A. (Intercepta) control	16.175545	1.07	15.10	-
A. 5 mg/kg	3.60	1.43	2.51	0.0123*
A.10 mg/kg	−1.74	1.89	−0.92	0.35
A.15 mg/kg^a^	0.17	1.37	0.12	0.90
MED. (Intercepta) control	10.37	0.60	17.26	-
MED. 5 mg/kg	−0.21	0.90	−0.23	0.81
MED. 10 mg/kg	0.58	1.21	0.47	0.63
MED. 15 mg/kg^a^	−3.20	0.84	−3.80	0.0002***
S. (Intercepta) control	2.79	0.42	6.65	-
S. 5 mg/kg	−0.18	0.64	−0.27	0.78
S. 10 mg/kg	0.64	0.76	0.84	0.39
S.15 mg/kg^a^	0.40	0.64	0.62	0.53

The data A (asymptote), MED (half-life) and S (scale) are correlated to their respective controls as designated in the table *Intercepta*. One individual in the PEG-PSL 15 mg/kg group died (a) one day after the booster. P-value < 0.05 (*) and < 0.0001 (***)

The coefficient half-life (the time it took the experimental groups to present an elevated disease score in half the total experiment time) demonstrated that the 15 mg/kg treatment rapidly achieved a high score, compared to the control (Table 2) and other treatments. One may postulate that these results are related to the two animals with scores 2 and 3 on day zero and the animal that died on the second day after the booster. Although this data could point to a possible toxic effect of the dose 15 mg/kg, the other animals in the same group followed a similar disease progression compared with other groups. Therefore, it may be that the animal that died had a heterogeneous manifestation of the disease following the CIA protocol.

The scale coefficient, which evaluates the slope of the curve determining disease progression, detected no significant difference between the treatments and the control.

Two of the six animals treated with 5 mg/kg presented delayed development of the disease, one on the 7th day after the booster, another animal only on the 13th day, affecting the incidence of the disease and the value of the upper asymptote.

### Histopathological analysis of the tibiotarsal joint of mice with collagen-induced arthritis (CIA) treated with PEG-PSL

**Fig. 2:**
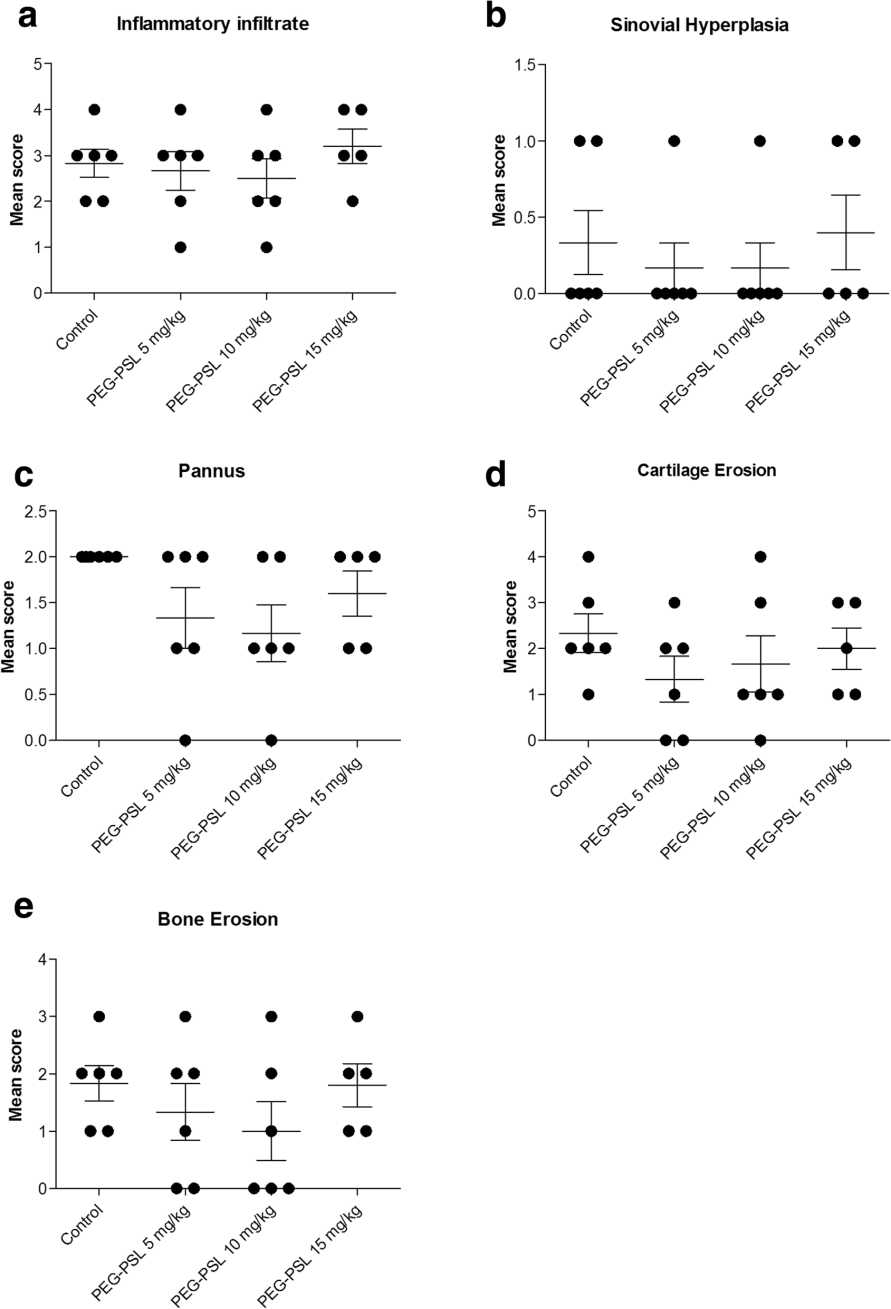
Histophathological analysis of the tibia-tarsal joint of mice. The tibiotarsal joints of the DBA/1J animals were evaluated for inflammatory infiltration (**a**), synovial hyperplasia (**b**), pannus formation (**c**), erosion of cartilage (**d**), and bone (**e**). Kruskal-Wallis test and Dunn’s Multiple Comparison were used. *P*-value < 0.05 (*)

The histology scores can be observed in graphic (Fig. 2). Treatment with different doses of PEG-PSL did not present any improvement regarding the histological parameters analyzed. The animals presented marked joint abnormality with pronounced inflammatory infiltration (Fig. 2a), synovial hyperplasia (Fig. 2b), extensive pannus formation (Fig. 2c), severe erosion of cartilage (Fig. 2d) and bone (Fig. 2e), independent of treatment, with no statistically significant difference between the groups analyzed. Regarding the deterioration of cartilage, analysis by Safranin-O staining (data not presented) showed that there was no reduction in this symptom by the treatment as compared to the control, indicating that PEG-PSL did not protect from cartilage deterioration.

#### Levels of TNF-α in the knee joints of the animals did not show any reduction in groups PEG-PSL 5, 10 and 15 mg/kg, compared to the control

**Fig. 3:**
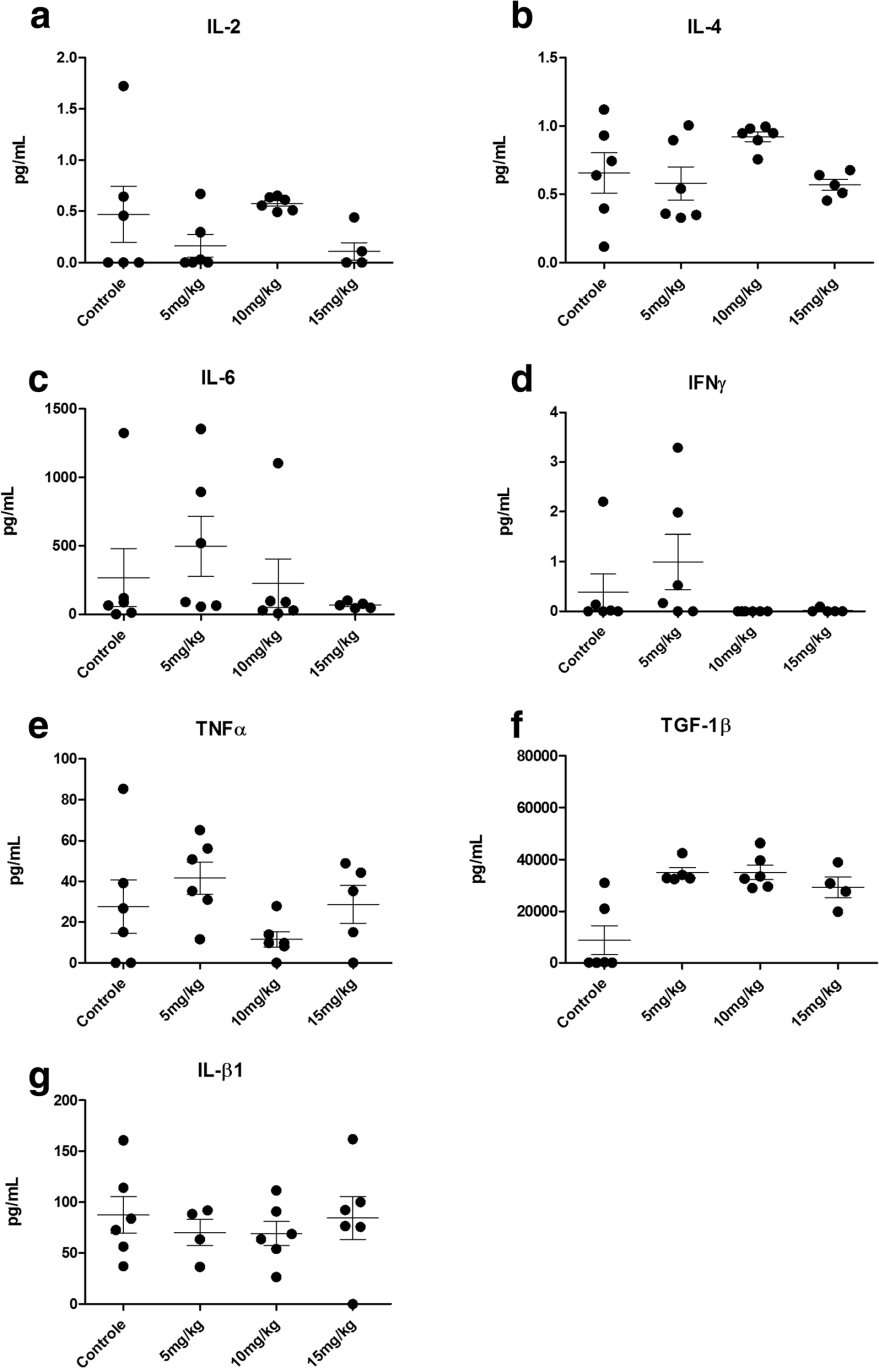
Cytokines evaluation of knee joints. CBA was used to evaluate **a** *,* IL-2*;* **b**, IL-4*;* **c**, IL-6*;* **d** *,* IFNγ and **e** *,* TNFα, whereas **f**, TGF-1β and **g**, IL-β1 were evaluated by ELISA. The measurement was performed on 20th day of treatment, by homogenization of the knee joints of the animals. For statistical analysis, the Kruskal-Wallis test was used first to determine which response any significant difference in response, followed by the unpaired Wilcoxon test, to determine if contrasts had a significant response. There was no significant difference observed. *P*-value < 0.05 (*)

The concentrations of TNF-α, IL-1β, IL-2, IL-6 and IFN-γ in the knee joints of the animals at the end of the experiment did not present significantly reduced levels in the PEG-PSL treated groups compared with the control (Fig. 3).

Concentrations of IL-17 and IL-10 were not present in detectable levels. Levels of TGF-β1 in the groups treated with PEG-PSL did not show significance relative to the control, however, analysis showed enhanced of levels in the PEG-PSL groups (Fig. 3f). Despite the lack of statistical significance between treated and control groups, the Kruskal-Wallis test indicated a significant difference of 5 % for the variable TNF-α (Fig. 3e), although the difference is related to the contrasts PEG-PSL 5, 10 and 15 mg/kg (Fig. 3e). However, in comparison to the control, group PEG-PSL 10 mg/kg showed no statistical significance.

### *In vitro*, PEG-PSL 100 μM did not inhibit the inflammatory process induced by LPS/PMA

In *in vitro* experiments using macrophages (RAW 264.7), there was no reduction of TNF-α, IL-6 per CBA in cultures stimulated with LPS/PMA when treated with PEG-LSP 100 μM.

**Fig. 4:**
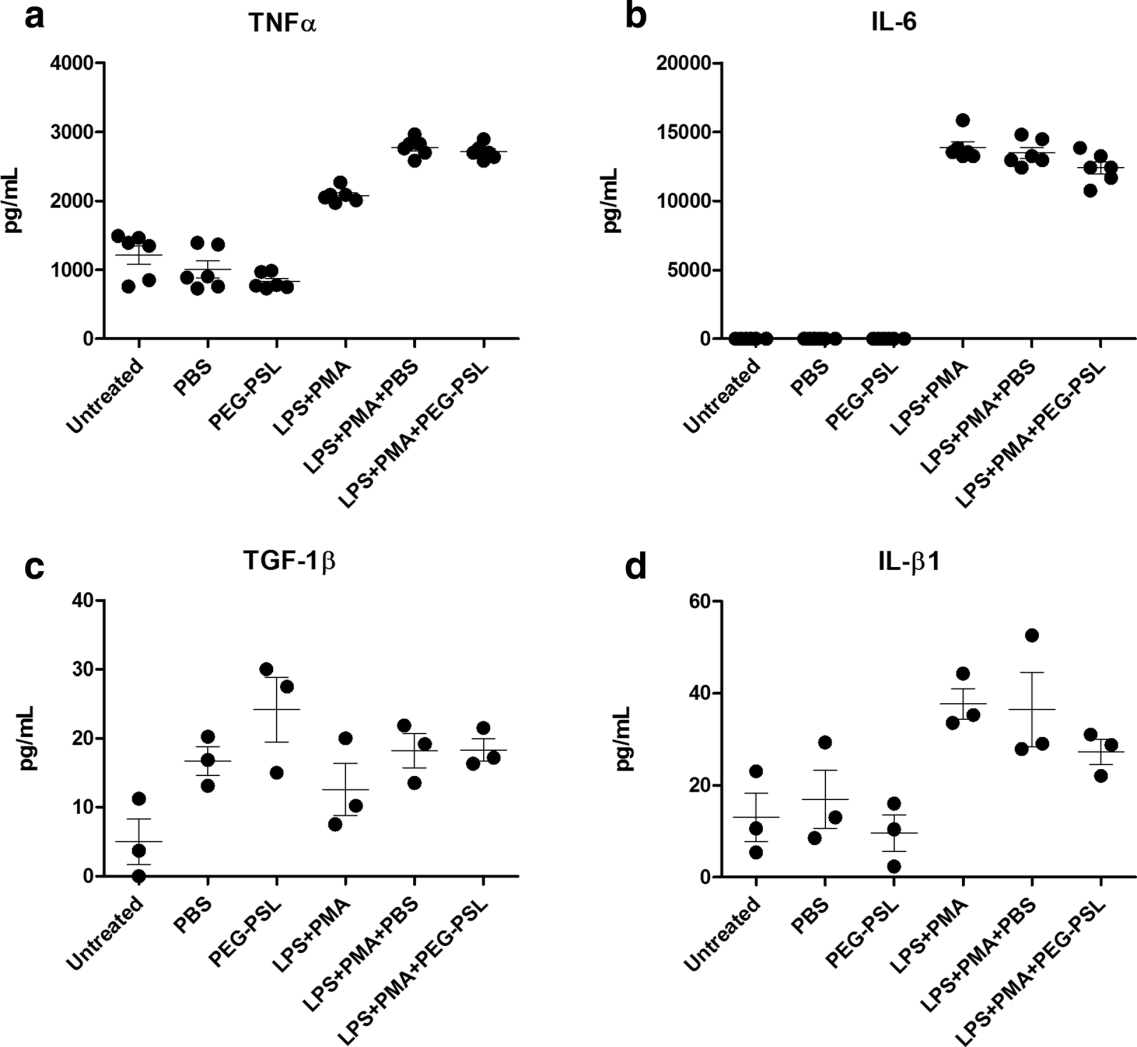
Cytokines evaluation of supernatant culture. CBA was used to evaluate TNFα (**a**) and IL-6 (**b**), whereas TGF-1β (**c**) and IL-β1 (**d**) were evaluated by ELISA. The culture supernatants were collected as described in the Table 1. The Kruskal-Wallis test was followed by the Wilcoxon unpaired *t*-test to determine if contrasts attained significant response. There was no significant difference observed. *P*-value < 0.05 (*)

The analysis by CBA did not allow detection of IL-2, IL-4, IFN-γ, IL-17 and IL-10. More sensitive methods should be used for this evaluation. The TGF-β1 evaluated by ELISA did not present significantly distinct levels between treated groups and the control (Fig. 4c).

The results clearly show the pro-inflammatory effect of LPS and PMA in the cultures (Fig. 4a, b and d). The Wilcoxon nonparametric *t*-test was again used to show any differences in the TNF-α response vis-à-vis contrast (treatment) (Fig. 4a).

The untreated culture groups, PBS and PEG-PSL 100 μM, showed significant difference regarding the expression of TNF-α in the supernatant compared to groups treated with LPS and PMA. PSL-PEG 100 μM did not achieve significant reduction of TNF-α compared to the untreated and PBS groups, though it was not toxic to macrophage culture (Fig. 4a).

Cultures stimulated with LPS and PMA, as expected, had elevated levels of TNF-α compared to non-stimulated groups, though none of the groups (LPS + PMA, LPS + PMA + PBS and LPS + PMA + PEG-PSL 100 μM) showed any significant difference in the concentration of TNF-α (Fig. 4a), demonstrating that PEG-PSL at a concentration of 100 μM did not reduce the pro-inflammatory effect induced by LPS and PMA.

For the IL-1β response (Fig. 4d), the Wilcoxon nonparametric *t*-test was also used to show any difference in the response vis-à-vis contrasts (treatments), however, the test showed no statistical difference between groups.

## Discussion

Apoptotic mimicking based on the use of PSL has been shown to reduce the inflammatory process in experimental models of acute arthritis [5, 12–14]. This study is the first of kind, evaluating the effect of administration of PEG-PSL (5, 10 and 15 mg/kg) in the experimental model of chronic arthritis (CIA), through analysis of clinical scores, levels of cytokines in the knee joints of the animals, disease incidence, histology of the paws and *in vitro* study using macrophages (RAW 264.7) stimulated with LPS/PMA.

The experiment setup has been designed to answer the main question: does PEG-PSL affect the inflammatory response trigged by CIA in DBA/1J mice?

As previously described, no significant differences between the untreated animals and those treated with PEG-PSL were detected regarding cytokine expression, clinical score, histological evaluation, and even in *in vitro* assays upon LPS/PMA stimulation.

The present experimental approach has shown some peculiarities compared with previous experiments, which certainly could be critical to the results presented.

Intramuscular administration of PSL offered some positive results in previous experiments conducted by Wu et al. [13] and Ma et al. [14] in adjuvant-induced arthritis (AIA) in rats. Mice, unlike rats, have a relatively tiny muscle mass in the thigh, therefore, the choice of subcutaneous administration for this study was based on the extreme difficulty of daily intramuscular administration of PSL, where the injection itself which would be likely to cause extreme pain and regional swelling.

The advantage of the intramuscular route is that PSL is rapidly absorbed, increasing the PSL concentration in the blood. Conversely, subcutaneous administration generally provides a slower absorption that could limit the systemic effect of PSL. In order to reduce PSL local response in the dermis, and increase a systemic response by enhancing concentration and time of PSL circulation in the blood, PEGylation was chosen as a viable alternative.

According to previous studies, incorporation of 2 mol% PEG in PSL does not affect the interaction of PS with macrophages and consequently PSL internalization, since at least 10–15 mol% of PEG would be needed to completely shield the liposomes from any interactions with proteins [29, 30]. Even 5 mol% PEG has been shown to have no effect on PSL-macrophages interaction [24]. However the need for studies evaluating the pharmacokinetics and accumulation of PEG-PSL in the periarticular tissue should be considered. The necessity of PEG-PSL in the inflamed region has not yet been evaluated by previous studies, but certainly, the route of administration has an important impact on the action of PEG-PSL.

The PSL dose used in our experiments is another critical factor that might affect the efficacy of PSL in CIA. Commonly used, 5 mg/kg of PSL has been shown to prevent ischemia in mice (5 mg/mg) [19], myocardial infarction in mice [23] and impact on AIA experimental model in rats [13, 14]. However, a carrageenan-induced arthritis model in mice used PSL 100 mg/kg via intraperitoneal [12], showed positive results. Therefore, it may be necessary to use a more sensitive titration assay and different administration routes to demonstrate any positive effects of PEG-PSL in future experiments in the CIA model.

## Conclusion

PEG-PSL, both in the dosage and the route of administration used in this study’s experimental model, showed no suppression of the chronic inflammation induced by CIA, even *in vitro* after stimulation of macrophages. However, the results herein indicate that perhaps the severe arthritis induced by CIA in our model, presenting high levels of TGF-β1 and other inflammatory cytokines in all the groups, could be an important variable to explore as to the mechanism of action of PEG-PSL, which is still not fully understood.

Further studies need to be conducted to evaluate the therapeutic intervention of PEG-PSL in the experimental CIA model and in the acute model, employing other alternatives than the dosage and route of administration. A new approach using PEG-PSL as a therapeutic adjuvant combined with a biological medicine with proven therapeutic efficacy can also be explored in future studies. New promoter routes that regulate synovial molecules must also be characterized to corroborate these PEG-PSL study findings as a new therapeutic tool in inflammatory diseases.

## Abbreviations

CBA: Cytometric bead array
CII: Bovine type II collagen
CIA: Collagen induced arthritis
DMEM: Dulbecco’s Modified Eagle Medium
ELISA: Enzyme-linked immunosorbent assay
CFA: Freund’s complete adjuvant
HMF: High-power magnification fields
IFA: Incomplete Freund adjuvant
PEG-PSL: Pegylated phosphatidylserine-containing liposomes
PMA: Phorbol myristate acetate
PSL: Phosphatidylserine-containing liposomes
RA: Rheumatoid arthritis
Sc: Subcutaneously
TIM: T cell immunoglobulin mucin receptors
VEGF: Vascular endothelial growth factor

## References

[1] Fadok VA, Voelker DR, Campbell PA, Cohen JJ, Bratton DL, Henson PM (1992). Exposure of phosphatidylserine on the surface of apoptotic lymphocytes triggers specific recognition and removal by macrophages. J Immunol.

[2] Savill J, Gregory C (2007). Apoptotic PS to phagocyte TIM-4: eat me. Immunity.

[3] Fadok VA, Bratton DL, Rose DM, Pearson A, Ezekewitz RA, Henson PM (2000). A receptor for phosphatidylserine-specific clearance of apoptotic cells. Nature.

[4] Fadok VA, de Cathelineau A, Daleke DL, Henson PM, Bratton DL (2001). Loss of phospholipid asymmetry and surface exposure of phosphatidylserine is required for phagocytosis of apoptotic cells by macrophages and fibroblasts. J Biol Chem.

[5] Hoffmann PR, Kench JA, Vondracek A, Kruk E, Daleke DL, Jordan M, Marrack P, Henson PM, Fadok VA (2005). Interaction between phosphatidylserine and the phosphatidylserine receptor inhibits immune responses in vivo. J Immunol.

[6] Seabra SH, de Souza W, Damatta RA (2004). Toxoplasma gondii exposes phosphatidylserine inducing a TGF-beta1 autocrine effect orchestrating macrophage evasion. Biochem Biophys Res Commun.

[7] Shaha C (2006). Apoptosis in Leishmania species & its relevance to disease pathogenesis. Indian J Med Res.

[8] Damatta RA, Seabra SH, Deolindo P, Arnholdt ACV, Manhães L, Goldenberg S, de Souza W (2007). Trypanosoma cruzi exposes phosphatidylserine as an evasion mechanism. FEMS Microbiol Lett.

[9] Wanderley JLM, Benjamin A, Real F, Bonomo A, Moreira MEC, Barcinski MA (2005). Apoptotic mimicry: an altruistic behavior in host/Leishmania interplay. Brazilian J Med Biol Res.

[10] Van Zandbergen G, Bollinger A, Wenzel A, Kamhawi S, Voll R, Klinger M, Müller A, Hölscher C, Herrmann M, Sacks D, Solbach W, Laskay T (2006). Leishmania disease development depends on the presence of apoptotic promastigotes in the virulent inoculum. Proc Natl Acad Sci U S A.

[11] Lima LG, Chammas R, Monteiro RQ, Moreira MEC, Barcinski MA (2009). Tumor-derived microvesicles modulate the establishment of metastatic melanoma in a phosphatidylserine-dependent manner. Cancer Lett.

[12] Ramos GC, Fernandes D, Charão CT, Souza DG, Teixeira MM, Assreuy J (2007). Apoptotic mimicry: phosphatidylserine liposomes reduce inflammation through activation of peroxisome proliferator-activated receptors (PPARs) in vivo. Br J Pharmacol.

[13] Wu Z, Ma HM, Kukita T, Nakanishi Y, Nakanishi H (2010). Phosphatidylserine-containing liposomes inhibit the differentiation of osteoclasts and trabecular bone loss. J Immunol.

[14] Ma HM, Wu Z, Nakanishi H (2011). Phosphatidylserine-containing liposomes suppress inflammatory bone loss by ameliorating the cytokine imbalance provoked by infiltrated macrophages. Lab Invest.

[15] Kobayashi N, Karisola P, Peña-Cruz V, Dorfman DM, Jinushi M, Umetsu SE, Butte MJ, Nagumo H, Chernova I, Zhu B, Sharpe AH, Ito S, Dranoff G, Kaplan GG, Casasnovas JM, Umetsu DT, Dekruyff RH, Freeman GJ (2007). TIM-1 and TIM-4 glycoproteins bind phosphatidylserine and mediate uptake of apoptotic cells. Immunity.

[16] Santiago C, Ballesteros A, Martínez-Muñoz L, Mellado M, Kaplan GG, Freeman GJ, Casasnovas JM (2007). Structures of T cell immunoglobulin mucin protein 4 show a metal-Ion-dependent ligand binding site where phosphatidylserine binds. Immunity.

[17] Taylor RC, Cullen SP, Martin SJ (2008). Apoptosis: controlled demolition at the cellular level. Nat Rev Mol Cell Biol.

[18] Huynh M-LN, Fadok VA, Henson PM (2002). Phosphatidylserine-dependent ingestion of apoptotic cells promotes TGF-beta1 secretion and the resolution of inflammation. J Clin Invest.

[19] Dvoriantchikova G, Agudelo C, Hernandez E, Shestopalov VI, Ivanov D (2009). Phosphatidylserine-containing liposomes promote maximal survival of retinal neurons after ischemic injury. J Cereb Blood Flow Metab.

[20] Ramani K, Miclea RD, Purohit VS, Mager DE, Straubinger RM, Balu-Iyer SV (2008). Phosphatidylserine containing liposomes reduce immunogenicity of recombinant human factor VIII (rFVIII) in a murine model of hemophilia A. J Pharm Sci.

[21] Ramani K, Purohit V, Miclea R, Gaitonde P, Straubinger RM, Balu-Iyer SV (2008). Passive transfer of polyethylene glycol to liposomal-recombinant human FVIII enhances its efficacy in a murine model for hemophilia A. J Pharm Sci.

[22] Gaitonde P, Peng A, Straubinger RM, Bankert RB, Balu-Iyer SV (2011). Phosphatidylserine reduces immune response against human recombinant Factor VIII in Hemophilia A mice by regulation of dendritic cell function. Clin Immunol.

[23] Harel-Adar T, Ben Mordechai T, Amsalem Y, Feinberg MS, Leor J, Cohen S (2011). Modulation of cardiac macrophages by phosphatidylserine-presenting liposomes improves infarct repair. Proc Natl Acad Sci U S A.

[24] Geelen T, Yeo SY, Paulis LEM, Starmans LWE, Nicolay K, Strijkers GJ (2012). Internalization of paramagnetic phosphatidylserine-containing liposomes by macrophages. J Nanobiotechnology.

[25] Trentham DE, Townes AS, Kang AH (1977). Autoimmunity to type II collagen an experimental model of arthritis. J Exp Med.

[26] Svensson L, Jirholt J, Holmdahl R, Jansson L (1998). B cell-deficient mice do not develop type II collagen-induced arthritis (CIA). Clin Exp Immunol.

[27] Brand DD, Latham KA, Rosloniec EF (2007). Collagen-induced arthritis. Nat Protoc.

[28] Oliveira PG, Grespan R, Pinto LG, Meurer L, Brenol JCT, Roesler R, Schwartsmann G, Cunha FQ, Xavier RM (2011). Protective effect of RC-3095, an antagonist of the gastrin-releasing peptide receptor, in experimental arthritis. Arthritis Rheum.

[29] Chiu GN, Bally MB, Mayer LD (2001). Selective protein interactions with phosphatidylserine containing liposomes alter the steric stabilization properties of poly(ethylene glycol). Biochim Biophys Acta.

[30] Levchenko TS, Rammohan R, Lukyanov AN, Whiteman KR, Torchilin VP (2002). Liposome clearance in mice: the effect of a separate and combined presence of surface charge and polymer coating. Int J Pharm.

